# SARS-CoV-2 Spike Protein Suppresses ACE2 and Type I Interferon Expression in Primary Cells From Macaque Lung Bronchoalveolar Lavage

**DOI:** 10.3389/fimmu.2021.658428

**Published:** 2021-06-04

**Authors:** Yongjun Sui, Jianping Li, David J. Venzon, Jay A. Berzofsky

**Affiliations:** ^1^ Vaccine Branch, Center of Cancer Research, National Cancer Institute, National Institutes of Health (NIH), Bethesda, MD, United States; ^2^ Biostatistics and Data Management Section, Center of Cancer Research, National Cancer Institute, National Institutes of Health (NIH), Bethesda, MD, United States

**Keywords:** SARS-CoV-2, lung bronchoalveolar lavage, spike protein, ACE2, type I interferon

## Abstract

SARS-CoV-2 virus causes upper and lower respiratory diseases including pneumonia, and in some cases, leads to lethal pulmonary failure. Angiotensin converting enzyme-2 (ACE2), the receptor for cellular entry of SARS-CoV-2 virus, has been shown to protect against severe acute lung failure. Here, we provide evidence that SARS-CoV-2 spike protein S1 reduced the mRNA expression of ACE2 and type I interferons in primary cells of lung bronchoalveolar lavage (BAL) from naïve rhesus macaques. The expression levels of ACE2 and type I interferons were also found to be correlated with each other, consistent with the recent finding that ACE2 is an interferon-inducible gene. Furthermore, induction of ACE2 and type I interferons by poly I:C, an interferon inducer, was suppressed by S1 protein in primary cells of BAL. These observations suggest that the downregulation of ACE2 and type I interferons induced by S1 protein may directly contribute to SARS-CoV-2-associated lung diseases.

## Introduction

SARS-CoV-2 virus, the causative agent of COVID-19, infects a wide array of cells, including epithelial cells, endothelial cells, and macrophages of multiple organs such as lung, gut, liver and kidneys *via* angiotensin-converting enzyme 2 (ACE2) as a receptor and transmembrane protease serine 2 (TMPRSS2) as an activating protease (1). In the human respiratory system, ACE2 and TMPRSS2 were primarily expressed in type II pneumocytes and a fraction of secretory cells (1–3). Human virologic and macaque viral challenge studies showed that SARS-CoV-2 virus can productively infect the target cells of upper and lower respiratory airways, which results in a quick peak viral load (2-3 days post viral challenge in macaques) in the lung bronchoalveolar lavage (BAL) fluid (4, 5). Infected patients displayed symptoms ranging from mild to severe pneumonia, and in some cases, acute respiratory distress syndrome (ARDS) or lethal pulmonary failure (6). However, the interplay between the lung immune microenvironment and SARS-CoV-2 virus or its viral components is unclear.

In this study, we focused on characterizing the alterations of ACE2 and type I interferons induced by viral spike protein S1 in the lung BAL fluids from naïve macaques. SARS-CoV-2 spike protein is a large type I transmembrane protein which is cleaved into two subunits, S1 and S2. S1 is responsible for recognizing the cell surface receptor ACE2 through a receptor binding domain (RBD), while S2 is needed for membrane fusion (7). ACE2 is a key component of the renin-angiotensin system, which cleaves angiotensin II to generate ang1-7 (8). Accumulated angiotensin II activates angiotensin II receptor type 1 (AT1R) in the lung and results in increased vascular permeability, immune cell infiltration, and lung edema (9). ACE2 has been shown to play important roles in respiratory virus infections, especially coronavirus infections. ACE2 mediated protection against lethal avian influenza H5N1 infection by reducing levels of angiotensin II (10). In an experimental mouse model of influenza H7N9 infection, ACE2 deficiency caused severe acute lung injury (11). Similarly, during the earlier SARS-CoV infection, the reduction of ACE2 expression contributed to acute lung failure through modulation of the renin-angiotensin system (12).

It has been reported that the expression levels of ACE2 played an important role in determining the outcomes of SARS-CoV-2 infections (13–16). During the early stage, lower level of ACE2 in the lung is beneficial for the host to control viral transmission and replication. However, if there was not enough ACE2 for a prolonged time, the lack of ACE2 led to the reduced conversion of angiotensin II into ang1-7, and the accumulated angiotensin II might lead to increased immune activation, and eventually lung disease. Given the possible dual roles of ACE2 to either mediate viral infection or protect against lung disease, we evaluated the ACE2 expression level on primary cells of lung BAL fluids after exposure to SARS-CoV-2 spike protein S1, the subunit that interacts with ACE2. We also assessed the changes in the expression levels of chemokines/cytokines, especially type I interferons. Type I interferon mediates an important innate immune response against viral infection by directly inhibiting viral replication. It also bridges innate to adaptive immunity for long-term protection against viral reinfections (17, 18).

## Materials and Methods

### BAL Sample Collection and Cell Type Characterization by Flow Cytometry Analysis

Animals were anesthetized and intubated before up to 10 mL/kg of sterile saline was instilled into the lungs. Suction was then applied to recover the instilled fluid. Usually up to 90% of the fluid instilled was recovered. The collected BAL fluid was then passed through a 100 µm cell strainer to remove big chuncks. BAL cells were collected for analysis or cryopreservation after spinning down. For flow cytometric analysis, the BAL cells were first incubated with Fc Receptor blocking reagent (Miltenyi Biotec), and then stained with viability dye (Invitrogen) and antibody mixtures including CD45- Alexa Fluor 700, CD3-PE-Cy7, CD4-BV605, CD8-BV800 (all from BD Pharmingen), CD14-BV450, CD16-BV711, HLA-DR-APC-Cy7, CD11b-PE-Cy5, and CD163-PerCP (all from Biolegend). Data acquisition was performed on an LSRII flow cytometer, and FlowJo software (Tree Star Inc.) was used for data analyses.

### S1 Protein and Poly I:C Treatment of BAL Samples

Fresh or cryopreserved BAL/PBMCs were thawed and resuspended at a concentration of 2-4 million cells/ml in serum free medium AIM® (ThermoFisher). 1µg/ml (T1) or 2µg/ml (T2) of SARS-CoV-2 (2019-nCoV) Spike S1 protein (molecular weight 116KD, Cat: 40591-V08H, Sino Biological, selected lot with measured endotoxin level: <0.001U/µg) was added to the cells. In the case of poly I:C treatment, 1μg/ml of poly I:C were added in the cell culture in the presence or absence of S1 protein. After 20 hrs of culture at 37°C, 5% CO_2_, supernatant was collected and frozen at -20°C for cytokine and chemokine measurements. Trizol was added to the cells for RNA isolation.

### Real-Time RT-PCR to Assess mRNA Expression Levels of ACE2 and Type I Interferons

After RNA isolation (Zymo research), an optimized mix of random hexamers and anchored oligo dT primers, which completely represents the 5’ to 3’ RNA sequence, was used for reverse transcription (SensiFAST™ cDNA Synthesis Kit from Bioline USA Inc). cDNAs were used for qPCR reactions to detect the relative expression levels of target genes. Taqman probe and primer sets for macaque ACE2, CCL5 and type I interferon subtypes including IFNα1, 2, 4, 6, 8, and 14 and IFNβ1 and GAPDH were obtained from ThermoFisher. The PCR mixture contained 1μl of primer/probe set for each gene, 2μl (or 8μl) of cDNA, and 10μl of 2X SensiFast probe kit (Bioline USA Inc.). An ABI 7500 was used to run each PCR with a program consisting of 2 min at 50°C, 10 min at 95°C, and then followed by 45 cycles of 15 sec at 95°C, and 1 min at 60°C. The comparative threshold cycle (Ct) method of relative quantitation was used to compare the relative mRNA expression levels (PerkinElmer User Bulletin no. 2). Housekeeping gene GAPDH was used for normalization.

### Multiplex and ELISA to Measure the Cytokine/Chemokine and Pan-IFN α Expression in the Supernatant of the Treated BAL Samples

LEGENDplex™ NHP Chemokine/Cytokine Panel (13-plex, Biolegend) and Pan-IFN α (including subtype α1, 2, 4, 5, 6, 7, 8, 10, 14, 16 and 17) ELISA kit (Mabtech) were used to evaluate the alteration of cytokines/chemokines and IFN α in the cell culture supernatant in accordance with the manufacturer’s instructions.

### Statistical Analysis

We performed statistical analyses with Prism version 8 (GraphPad Software). Wilcoxon signed rank tests were conducted to compare differences in outcomes with paired measurements. Spearman analysis was used for correlations. Qlucore Omics Explorer software was used for heatmap and PCA analysis.

## Results

### SARS-CoV-2 Spike Protein Suppressed ACE2 and Type I Interferon mRNA Expression in Most of the Primary Lung Bronchoalveolar Lavage Samples

To characterize the cell types in lung bronchoalveolar lavage (BAL) fluid, we obtained fresh samples from 9 naïve healthy Indian rhesus macaques and analyzed the stained samples with lineage immune markers *via* flow cytometry (**Supplementary Figure 1**). Among the live cells, about 60% (±7) were in the CD45 negative population, non-hematopoietic cells. Among the CD45^+^ populations (hematopoietic cells), while CD14^+^ and CD16^+^ monocytes constituted only about 1.6±0.2%, and 2.9±0.4% respectively, the majority of cells were CD3^+^ T cells (72.2±1.7%), with predominately CD8^+^ T cells (54 ±2%), CD4^+^ T cells (34±3%), and a small percent of double positive T cells (4±1%) (**Supplementary Figure 1**). In the non-T and non-monocyte CD45^+^ cell population, 18±5% were DR^+^CD11b^-^, and 5±2% were CD11b^+^DR^-^.

To evaluate the innate immunity induced by SARS-CoV-2 spike protein S1, we incubated the lung BAL cells with either 1 µg/ml (T1, 8.6nM) or 2 µg/ml (T2, 17.2nM) of S1 protein for 20 hours. We chose these two concentrations of S1 protein based on that 1) as high as 28nM of S protein was added for *in vitro* stimulations in a SARS *in vitro* study (19); 2) In SARS-CoV-2 and HIV studies, 1-5ug/ml (8-40 nM) spike/envelope protein (peptide pool) is used to stimulate antigen-specific cells *in vitro (* 20–24). This range is based on the viral load, and viral protein concentration *in vivo*. For SARS-CoV-2, the recent macaque virology data showed that the viral load in the infected lung is log 6-8, similar or higher than HIV infection. Therefore, both T1 and T2 concentrations are within physiological/pathological range. S1 protein did not cause significant cell death measured by LDH release (data not shown). When cytokine and chemokine multiplex assays were run on the collected supernatants, we did not observe significant induction of most chemokines or cytokines in the BAL treated with 1 or 2 µg/ml of S1 protein. Since the numbers of the primary cells collected from BAL cells of each animal varied, most of the samples were just enough for control and T1, and only several of them were enough for T2. Out of the 13 chemokines/cytokines measured at the protein level, only RANTES/CCL5 showed a trend toward an increase (**Supplementary Figure 2**).

**Figure 1:**
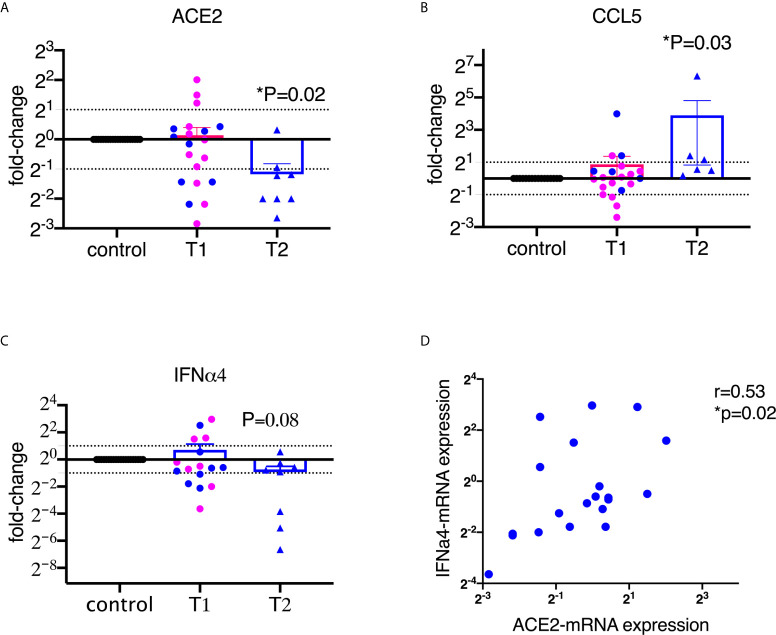
SARS-CoV-2 spike protein suppressed the mRNA expression of ACE2 and type I interferon in lung bronchoalveolar lavage from naïve macaques. Primary lung bronchoalveolar lavages from naïve rhesus macaques were cultured with 1 (T1) or 2 (T2) µg/ml of spike protein S1 for 20 hrs (n=20 and n=6 or 8, respectively). After the supernatants were collected, the cells were lysed with trizol and RNAs were isolated. After reverse transcription, macaque-specific primer/probe sets were used to measure the mRNA expression levels of ACE2, CCL5, and interferon α4 **(A–C)**. Data are shown as mean±SEM. Each dot presents one animal. The blue color indicates the samples from T1 that have corresponding cultures from matched animals in T2. Wilcoxon signed rank tests were used to calculate the p values. **(D)** ACE2 positively correlated with interferon α4 in lung bronchoalveolar lavage. Spearman R and p values are shown.

To assess the alteration of ACE2 expression upon encounter with SARS-CoV-2 spike protein S1 in the BAL samples, we isolated RNA and ran qPCR using macaque-specific primers and probes. As shown in **Figure 1A**, ACE2 mRNA levels were significantly decreased after treatment with 2 µg/ml of S1 protein for 20 hours, but not significantly at 1 µg/ml of S1 protein. It is worth mentioning that the downregulation of ACE2 by S1 could not be modulation off the surface because we are measuring mRNA, not surface protein. The mRNA for RANTES/CCL5 was significantly increased, consistent with the expression at the protein level (**Figure 1B**).

We then measured type I interferons, which play important roles in controlling viral infections (25). Most viral infections induce interferons, especially type I interferons. Here we assessed 6 subtypes of α interferons (α1, 2, 4, 6, 8, and 14) and interferon β1, and found that S1 protein did not induce robust α or β interferons in the BAL (**Supplementary Figure 3**). Moreover, some of the type I interferons, such as IFNα4, tended toward downregulation compared to the control at the concentration of 2 µg/ml of S1 protein (**Figure 1C**). A recent study demonstrated that ACE2 is an interferon-inducible gene (2). Indeed, our data also confirmed that ACE2 positively correlated with α2 and α4 interferon and showed a trend for α1 interferon (**Figure 1D**, **Supplementary Figure 4**).

**Figure 2:**
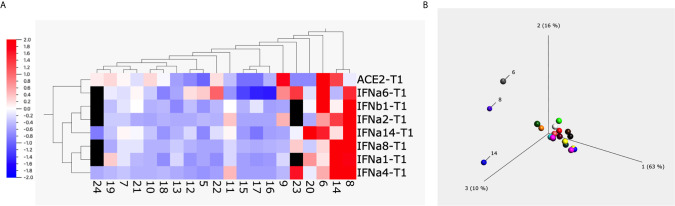
The heatmap and principal component analysis (PCA) plot of ACE2 and type I interferons after spike protein treatment. Heatmap **(A)** and PCA plot **(B)** were built up using the data obtained from BAL fluids of 20 naïve macaques treated with 1µg/ml of spike protein S1 for 20 hrs using Qlucore Omics Explorer software. The missing data (due to the low copy number of some genes) was filled with black squares. The scale in **(A)** is the relative expression level of the genes. The numbers below the heatmap, and in the PCA plot are the animal IDs.

To have a global picture, we used a heatmap and a principal component analysis (PCA) plot to visualize the alterations of different subtypes of type I interferons and ACE2 from 20 animals upon treatment with 1 µg/ml of S1 protein (**Figures 2A, B**). Both heatmap and PCA plot clearly showed the altered pattern induced by S1 treatment: most animals overall showed somewhat decreased expression of type I interferons and ACE2 at the mRNA level, whereas the unusual increase in ACE2 and type I interferons were mainly observed in just 3 animals, namely # 6, 8, and 14.

### SARS-CoV-2 Spike Protein Suppressed Poly I:C-Induced ACE2 and α4 Interferon Expression in Primary Lung Bronchoalveolar Lavage Samples

**Figure 3:**
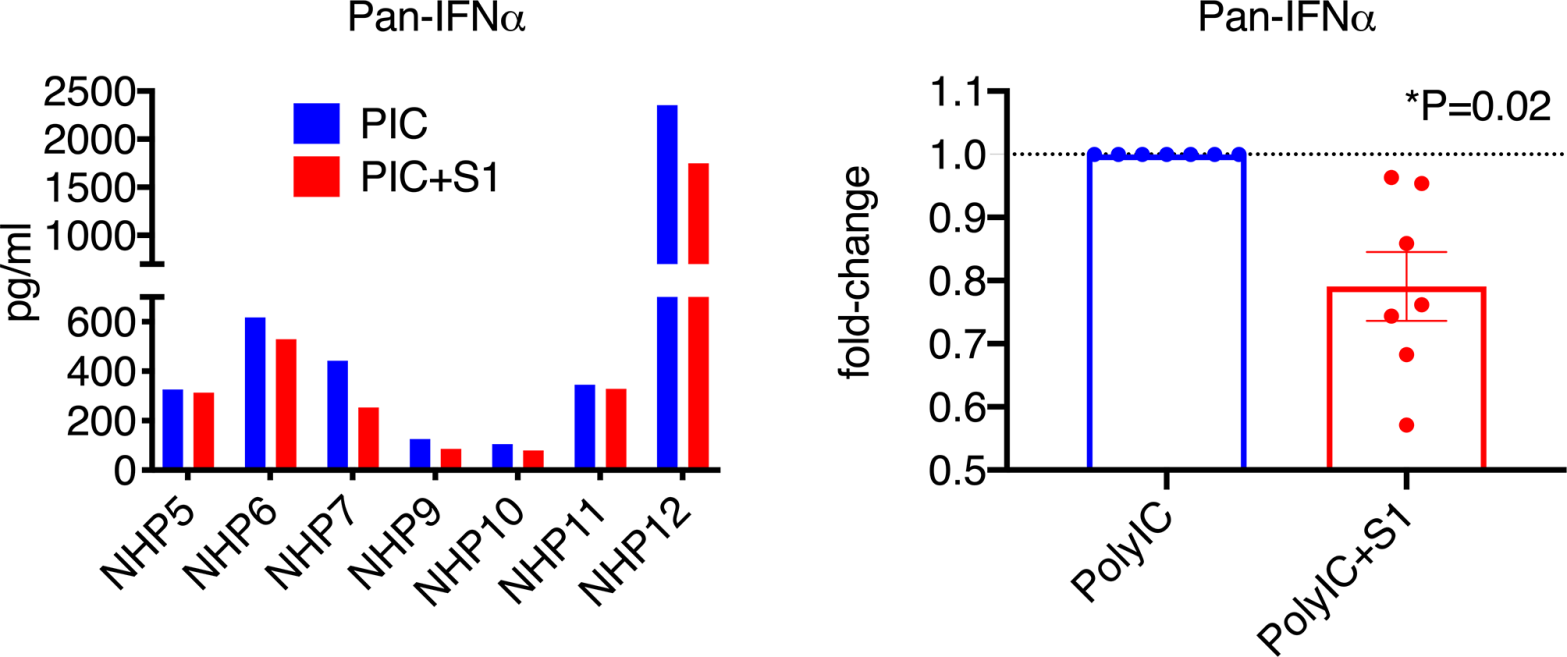
SARS-CoV-2 spike protein reduced poly I:C-induced interferon α expression in lung bronchoalveolar lavage from naïve macaques. Primary lung bronchoalveolar lavage fluids from 7 naïve rhesus macaques were cultured with 1 mg of poly I:C with or without 1 µg/ml of spike protein S1 for 20 hrs. Supernatants were collected, and a Pan interferon α ELISA was used to measure the total expression level of interferon α. The right panel shows the data with poly I:C normalized to 1 for each animal to calculate the fold changes in the presence of spike protein S1.

Since naïve BAL samples have only baseline, low levels of type I interferons, to better assess the effect of S1 protein, we co-treated BAL samples with poly I:C, which has been shown to induce high levels of type I interferons, and possibly ACE2, *via* the TLR3 receptor. Since interferon α has many subsets, we first measured the pan interferon α at the protein level. Without poly I:C, the pan interferon α protein level in the BAL samples from naïve animals was below or close to the detection limit. After co-culture with poly I:C, varied levels of (100-2500 pg/ml) pan interferon α were induced in BAL (**Figure 3**). Consistent with **Figure 1**, S1 protein significantly decreased poly I:C-induced pan interferon α at the protein level by about 25% (**Figure 3**), suggesting that S1 protein suppressed type I interferon expression in BAL.

**Figure 4:**
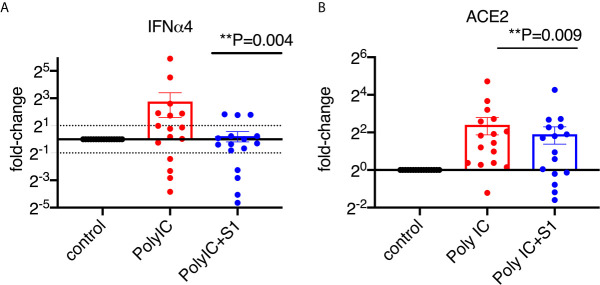
SARS-CoV-2 spike protein reduced poly I:C-induced mRNA expression of ACE2 and type I interferon in lung bronchoalveolar lavage from naïve macaques. Primary lung bronchoalveolar lavage fluids from 16 naïve rhesus macaques were cultured with 1 μg of poly I:C with or without 1 µg/ml of spike protein S1 for 20 hrs. RNAs were isolated with Trizol and followed by reverse transcription. Macaque-specific primer/probe sets for ACE2 **(B)**, interferon α4 **(A)** was used. Data are shown in mean±SEM. Wilcoxon signed rank tests were used to calculate the p values between the poly I:C-only fold change *vs* the poly I:C+S1 fold change for interferon and ACE2.

**Figure 5:**
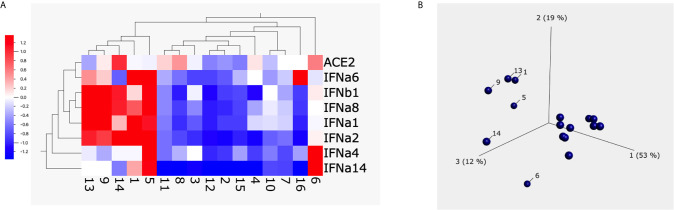
The heatmap and PCA plot of ACE2 and type I interferons after spike protein treatment. Heatmap **(A)** and PCA **(B)** were built up using Qlucore Omics Explorer software. The numbers below the heatmap and in the PCA plot are the animal IDs. Each dot presents one animal. The scale in **(A)** is the relative expression level of the genes.

To characterize the type I interferon subtypes, we used macaque-specific primers/probe sets for interferon α and β1 mRNA. In agreement with the expression at the protein level, poly I:C induced significantly increased type I interferons at the mRNA level compared to non-poly I:C treated samples (**Figure 4A**, **Supplementary Figure 5**). Poly I:C also induced significant expression of ACE2 (**Figure 4B**). We then evaluated whether 1 µg/ml of S1 protein could suppress the expression of type I interferons and ACE2 in the presence of poly I:C. Similar to the cells without poly I:C (**Figure 1**), ACE2 and α4 interferon induced by poly I:C were significantly decreased in the presence of 1 µg/ml of S1 protein, while the rest of the type I interferons measured in this study, including α1, 2, 6, 8, 14 and β1, did not change (**Figure 4A**, **Supplementary Figure 5**). We then calculated the ratios between treatment with S1 and without S1 for ACE2 and each interferon subtype. Based on these ratios, we found that 10 out of 16 animals had overall trends towards reduced type I interferon expression at the mRNA level in the presence of both S1 protein and poly I:C compared to poly I:C-only treatment, while only the remaining 6 animals showed some upregulation trends of type I interferons. Using these ratios, we generated the heatmap/PCA plot. The heatmap demonstrated that the outlier animals that had higher ratios of type I interferons were primarily just #1, 5, 9, 13, 14, and 6 (**Figure 5A**). A PCA plot confirmed the separation of the two groups of animals as well, with one group that showed downregulated trends of type I interferon and ACE2, and the other groups that did not (animal # 1, 5, 9, 13, 14 and 6, marked in **Figure 5B**). It is not clear what the underlying cause was of these different responses, as all the animals were naïve macaques.

Different mechanisms of type I interferon and ACE2 induction by poly I:C seemed to be possibly involved in the presence or absence of S1 protein. In the presence of poly I:C but without S1 protein, the expression of ACE2 and type I interferons did not correlate with each other except for the α6 subtype (**Supplementary Table 1**). However, after treatment with polyI:C and S1 protein, the mRNA expression levels of ACE2 did correlate with α1, 4, 6, and 14 subtypes of interferons (**Supplementary Tables 1, 2**). This suggested that S1 protein might have altered the cell signaling of ACE2 and type I interferon.

We did a correlation analysis to assess whether different compositions of the cell types in the lung played any roles in affecting the expression levels of type I interferon and ACE2. We found that the animals that demonstrated greater reduction of IFN alpha4 and ACE2 had higher levels of CD3^+^ T cells and CD11b^+^ myeloid cells in the lung (r= - 0.78, and - 0.77 separately, n=4). Even if the animals were naïve, the pre-treatment levels of T and myeloid cells in the lung may determine their responding directions. However, since the number of animals (n=4) is small, we cannot draw definite conclusions. Nevertheless, this is an interesting hypothesis that is worth testing in the future.

In summary, our data showed that S1 protein suppressed the expression of ACE2 at the mRNA level and decreased the mRNA expression of type I interferon, especially α4 interferon, induced by poly I:C in the primary BAL cells, as well as the level of secreted type I interferon protein.

## Discussion

During the course of SARS-CoV-2 infection, how the lung microenvironment is affected by viral spike protein is largely unknown. Because binding of spike protein to the ACE2 receptor on cells can serve not only to allow viral entry but also to trigger an effect on the cells, we asked whether spike-ACE2 signal might affect the innate immune response and trigger either a protective effect or a deleterious response that contributes to immunopathology. Using primary cells from macaque lung BAL, we demonstrated that in most of the animals, spike protein S1 directly caused downregulation of mRNA expression of viral receptor ACE2, and type I interferons especially α4 interferon. This has important implications for future therapeutic strategies for COVID19- related lung diseases, as well as mechanisms of COVID-19 immunopathology.

ACE2 is most highly expressed in lung and intestinal epithelial cells (26), and endothelial cells, and it might play dual roles in SARS-CoV-2 infections. As a receptor for the virus’s binding to and entry into the cells, the expression level of ACE2 might determine the viral transmission and replication efficacy. Because the binding affinity of SARS-CoV-2 spike protein to ACE2 is much higher than that of the earlier SARS-CoV spike protein, the target cells might not need a high density of ACE2 to be infected (1, 27). The kinetics of viral replication in SARS-CoV-2-infected humans and macaques showed that the peak viral loads appeared at day 2 post-infection, which is much earlier than most viral infections even for other viruses in the coronavirus family (4, 28, 29). Since only a small portion of the epithelial cells have ACE2 receptors (1, 3), SARS-CoV-2 infection in most cases is self-limiting. After day 2 peak viremia, the viral load starts declining with time in the lung, and most of the animals controlled their viral loads about 7-14 days post-infection (4, 28, 29). However, this does not mean that the damage to the host is diminished. Instead, the decreased expression of ACE2 we have discovered to be induced by spike protein may be more detrimental to the host. ACE2 protected animals from developing lung diseases in several viral infection models such as influenza H7N9 virus and respiratory syncytial virus (9, 12, 30, 31). Several animal studies have shown that downregulation or loss of ACE2 promotes lung injury (9, 12, 32). It has been reported that symptoms of lung injury, such as increased vascular permeability, lung edema, and neutrophil infiltration, were observed in an ACE2 knockout mouse model (33). These adverse effects are similar to those in the patients infected with SARS-CoV-2 virus. Given the protective role of ACE2 against lung diseases, ACE2 might act as a double-edged sword in SARS-CoV-2 infection. Despite these findings with respect to ACE2 expression and lung injury, pharmacological ACE2 blockade [e.g., by angiotensin II receptor blockers (ARBs)] is not correlated with clinical outcome, suggesting more studies are needed (34, 35). Our study showed that S1 protein of SARS-CoV-2 directly suppressed ACE2 expression in most of the BAL specimens, which suggested that spike protein S1 might dampen the ACE2-mediated tissue protective responses. The downregulation of ACE2 induced by spike protein in the lung could contribute to increased vascular permeability, lung edema, and massive infiltration of neutrophils and macrophages. If not resolved promptly, the patients may suffer more from inflammatory responses due to the “leaky lung” than from the viral load itself. This is similar to the previous observations on other viral respiratory diseases. The severity of lung disease was not directly correlated with viral loads but correlated with inflammatory responses (36, 37). Thinking along this line, opportunistic infections may contribute to promote inflammation after the increased lung vascular permeability due to the downregulation of ACE2. Further studies on the alterations of lung microbiome in COVID-19 patients might provide a mechanistic explanation of why massive inflammatory responses occurred in some of the infected patients. The downregulation of ACE2 by viral infection and/or S1 protein could be one of the mechanisms of recruitment of inflammatory cells to the lung, which promoted pulmonary fibrosis and ARDS in COVID-19 patients (13, 38, 39).

The downregulation of ACE2 upon S1 protein treatment could be due to the following reasons. After virus or viral protein interacts with ACE2 on target cells, the internalization of the ACE2 proteins on the cell surface might lead to negative feedback of the ACE2 expression. We speculated that pre-transcriptional regulation, such as histone modification and methylation, might be involved upon the stimulation of SARS-CoV-2 virus or spike protein. ACE2 expression in the lung could be regulated by enzymes that modify histones, including HAT1, HDAC2, and KDM5B (40). In both freshly isolated airway epithelial cells and human lung tissues, different patterns of *ACE2* gene methylation near the transcription start site of the ACE2 gene were identified in populations with different age and gender (41). MicroRNAs (miRNAs) could be involved in decreasing ACE2 expression at mRNA levels as well. The predominant mechanism of miRNAs inhibiting target gene expression is to destabilize the target mRNA, which accounts for most of the decreased protein production (42). About 2000 miRNAs that participated in the regulation of ACE2 have identified recently and could play a role in ACE2 downregulation after encountered with SARS-CoV-2 virus or S1 (43). Moreover, studies showed that no robust induction of type I interferons occurred after SARS-CoV-2 infection (44–46). Since ACE2 is an interferon-inducible gene (2), lack of type I interferon induction can lead to the regulation of ACE2 expression at the mRNA transcription level. Indeed, for that reason, downregulation of type I interferons like alpha4 interferon by spike protein could reduce ACE2 mRNA expression indirectly in addition to the direct effect.

After cellular detection of viral entry into a host cell, interferons and interferon-inducible genes are essential for host antiviral defense (17, 47–49). Our data showed that the majority of primary cells from different macaque BAL specimens reduced their expression of ACE2 and type I interferons, especially interferon α4, after exposure to S1 protein. Different pathogens induce a different profile of IFN-α subtypes, which leads to a qualitatively different immune response (50). For example, during influenza A virus infection, levels of interferon α4 and β1 mRNAs in the lungs of infected mice were elevated in the absence of PA-X (51), while HIV infected individuals transiently enhanced the expression of IFNα4, α5, α7, and α14 (52). Different subtypes of type I IFNs have overlapping but nonredundant roles and have distinct regulation mechanisms in various viral infections. Many studies have reported that as different cell types sensed viruses differently, distinct type I IFN subtype profiles were induced (53). Moreover, the amount of virus can also affect the subtypes of type I IFN subtypes induced due to the extent of activation of certain signaling pathways (54).

The mechanism by which S1 of SARS-CoV-2 inhibits type I interferon production is elusive. However, several mechanisms have been proposed and demonstrated in the studies on SARS-CoV and MERS-CoV viruses, which could be applied to S1 of SARS-CoV-2 as well. For example, MERS-CoV virus represses histone modifications, which downregulate expression of interferon-stimulation genes (55), while SARS-CoV infection induced type II interferon reduction was due to a step between the nuclear transport of IRF-3 and its subsequent activation by hyperphosphorylation and dimerization (56). Nucleocapsid protein (N protein) of SARS-CoV has been reported to suppress type I and III interferon production *via* interfering with TRIM25-mediated RIG-I Ubiquitination (57).

Type I interferons are important innate mediators of anti-viral immunity. SARS-CoV-2 has been shown more sensitive to type I interferon treatment compared to SARS-CoV in *in vitro* studies (58, 59). Furthermore, autoantibody against type I IFN and defects of type I IFN genes leads to life-threatening COVID-19 disease, suggesting the protective role of type I interferon (60, 61). Our finding that SARS-CoV-2 spike protein suppresses ACE2 and type I interferon expression in macaque BALs highlights the potential protective role in combating SARS-CoV-2 and strengthens the basis for using type I IFNs as therapies in COVID-19 patients. In light of the fact that ACE2 is an interferon-inducible gene, the concern that interferon-induced ACE2 might promote viral replication needs to be addressed. Onabajo et al. recently showed that what interferons induce is not the full length ACE2, but a primate-specific isoform of ACE2, which lacks 356 N-terminal amino acids and is non-functional in binding the SARS-CoV-2 spike protein (62).

Collectively, our study using primary cells from non-human primate BAL specimens indicates that spike protein S1 directly suppresses the mRNAs for ACE2 and type I interferons in most of the cases as well as secreted IFNα protein. This effect of S1 could explain the recent finding that type I interferon activity is diminished in severe COVID-19 infections (63). These findings have important implications in future therapeutic strategies to combat COVID-19 and related lung diseases and prevent associated pathologies.

## Data Availability Statement

The original contributions presented in the study are included in the article/**Supplementary Material**. Further inquiries can be directed to the corresponding authors.

## Author Contributions

YS and JB designed the projects. YS and JL performed and interpreted experiments. YS and DV performed statistical analyses. YS and JB wrote the manuscript with input from all the authors. All authors contributed to the article and approved the submitted version.

## Funding

This work was supported by the Intramural Program of the National Institutes of Health, National Cancer Institute, Center for Cancer Research funding Z01 SC 004020 to JB.

## Conflict of Interest

The authors declare that the research was conducted in the absence of any commercial or financial relationships that could be construed as a potential conflict of interest.
