# Supplementary material for: SARS-CoV-2 Spike Protein Suppresses ACE2 and Type I Interferon Expression in Primary Cells From Macaque Lung Bronchoalveolar Lavage
Source: Front Immunol. 2021 Jun 4;12:658428. doi: 10.3389/fimmu.2021.658428 (PMC8213020; doi:10.3389/fimmu.2021.658428)
Supplement: Supplementary file 1 [file DataSheet_1.pdf]

Supplementary Figure 1. Characteristic analysis of cell types in lung bronchoalveolar lavage from naïve healthy macaques

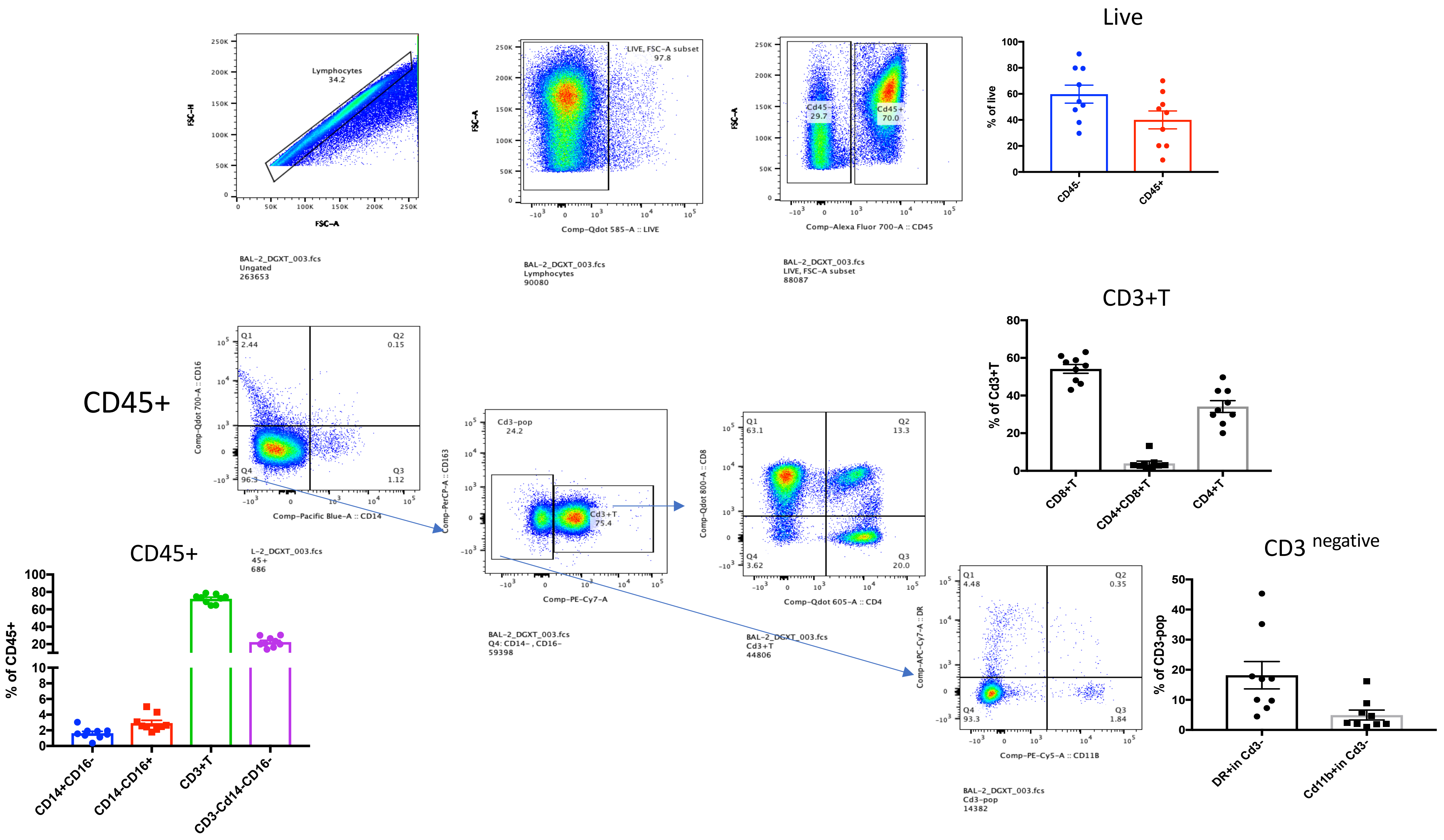

Supplementary Figure 2. SARS-CoV-2 spike protein treatment did not change the expression levels of most chemokines/cytokines secreted in lung bronchoalveolar lavage fluids at the protein level, except for RANTES/CCL5.

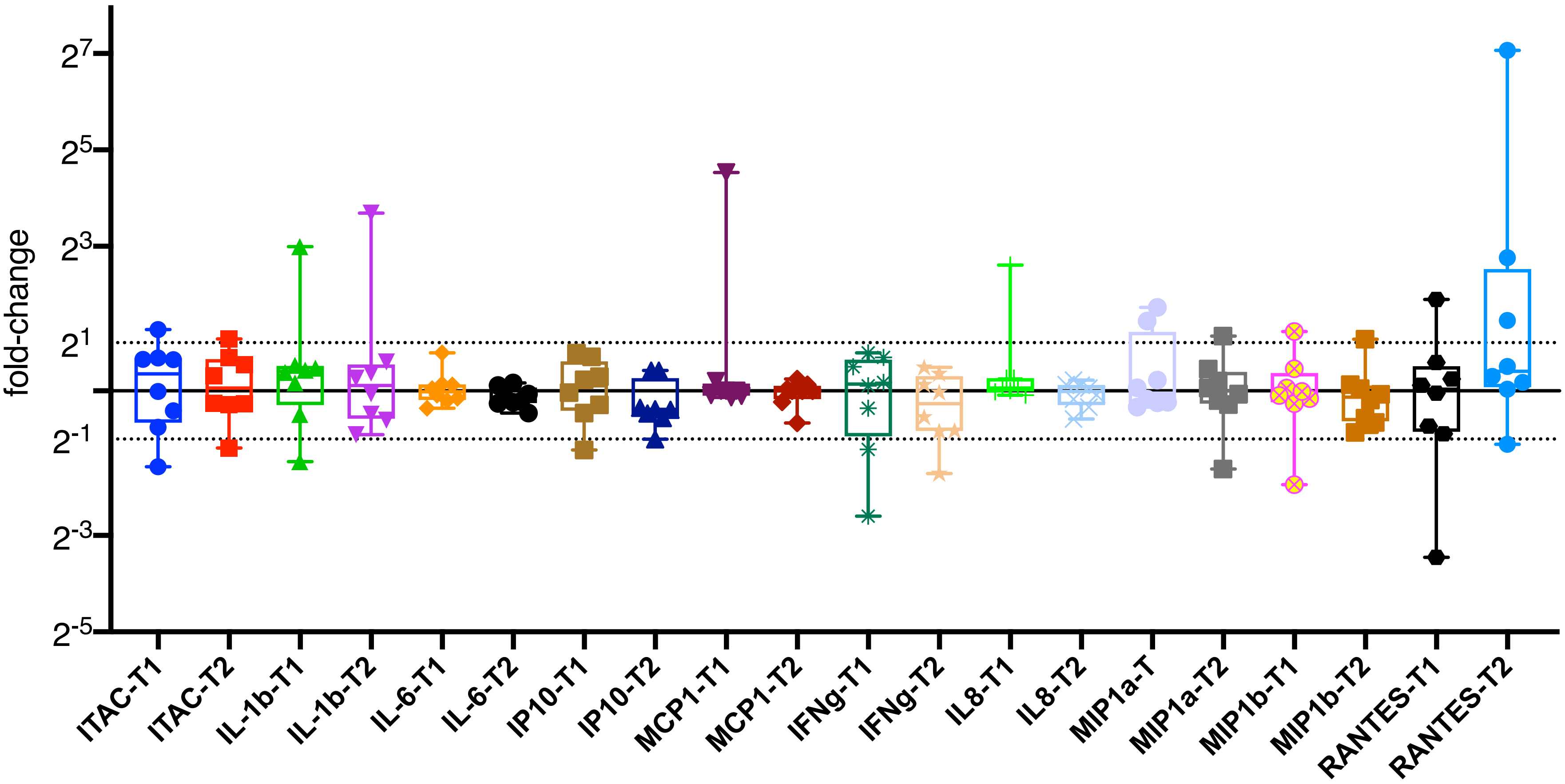

Box and whiskers: min to max

Supplememtary Figure 3. SARS-CoV-2 spike protein suppressed the mRNA expression of type I interferon in lung bronchoalveolar lavage from naïve macaques. Primary lung bronchoalveolar lavages from naïve rhesus macaques were cultured with 1 (T1) or 2 (T2) μg/ml of spike protein S1 for 20 hrs (n=20 and n=6, respectively). After the supernatants were collected, the cells were lysed with trizol and RNAs were isolated. After reverse transcription, macaque-specific primer/probe sets were used to measure the mRNA expression levels of ACE2, interferon 1, 2, 6, 8, 14 and 1,

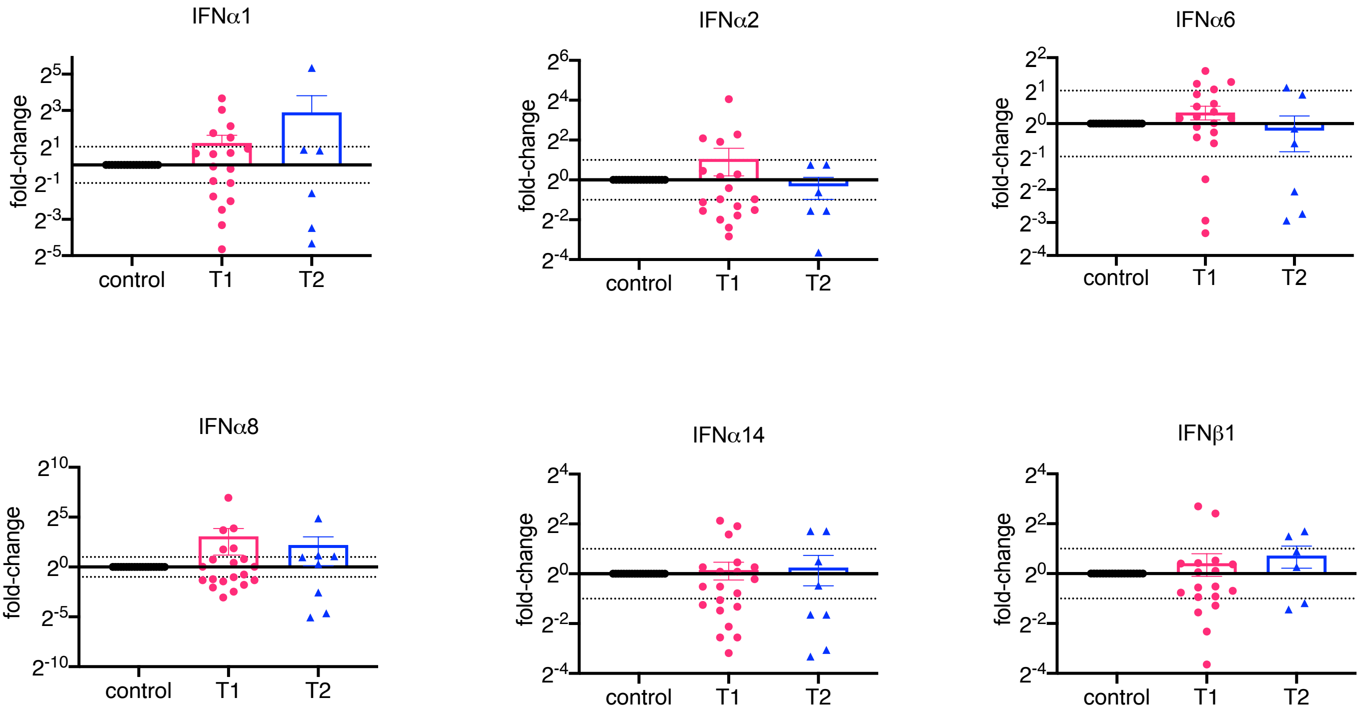

Supplementary Figure 4. ACE2 expression levels positively correlated with some subsets of type I interferons in lung bronchoalveolar lavage

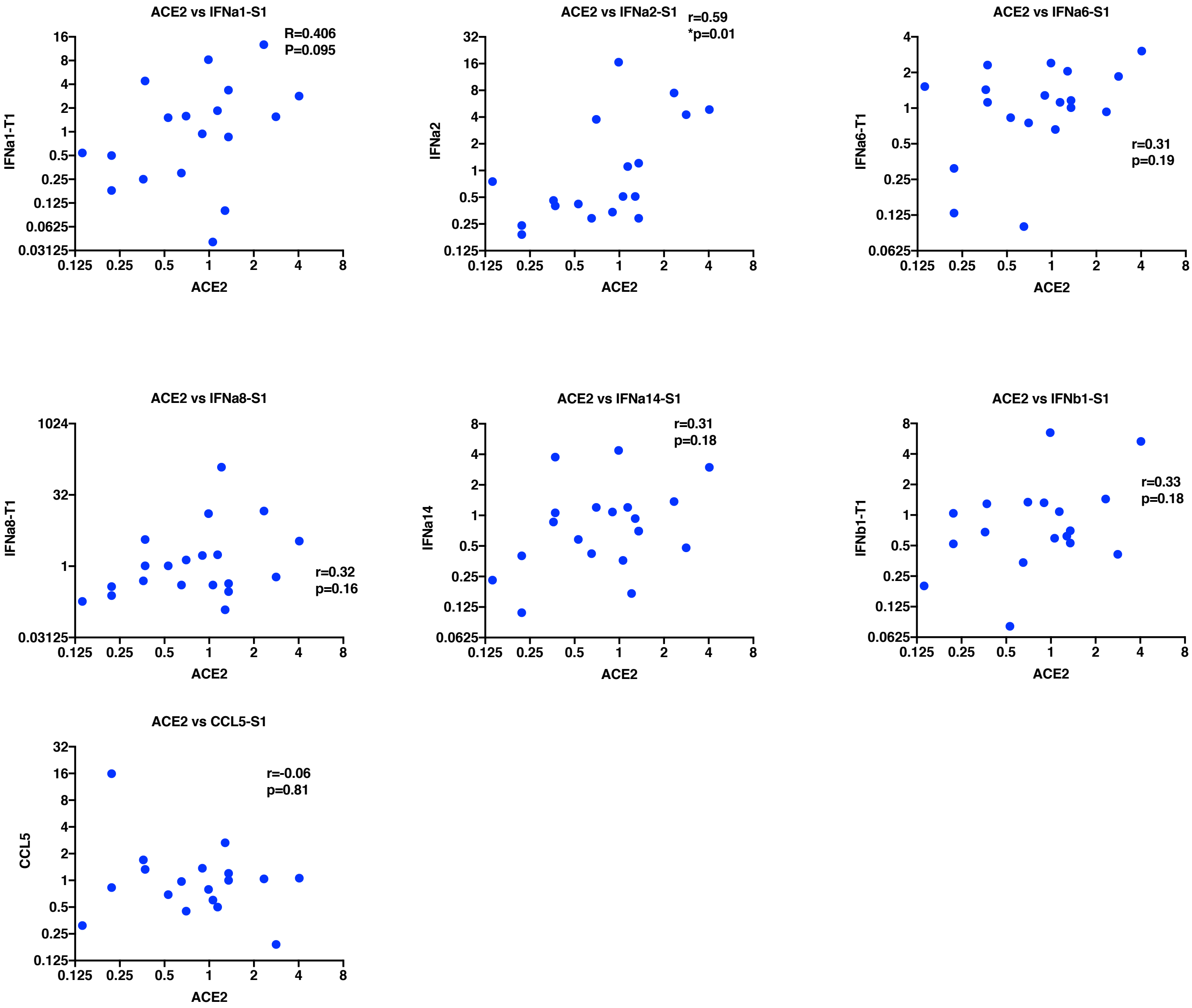

Supplementary Figure 5. SARS-CoV-2 spike protein reduced poly I:C-induced the mRNA expression of type I interferon in lung bronchoalveolar lavage from naïve macaques. Primary lung bronchoalveolar lavage fluids from 16 naïve rhesus macaques were cultured with 1 mg of poly I:C with or without 1 μg/ml of spike protein S1 for 20 hrs. RNAs were isolated with Trizol and followed by reverse transcription. Macaque-specific primer/probe sets for interferon α1, 2, 6, 8, 14 and 1were used. Data are shown in mean±SEM.

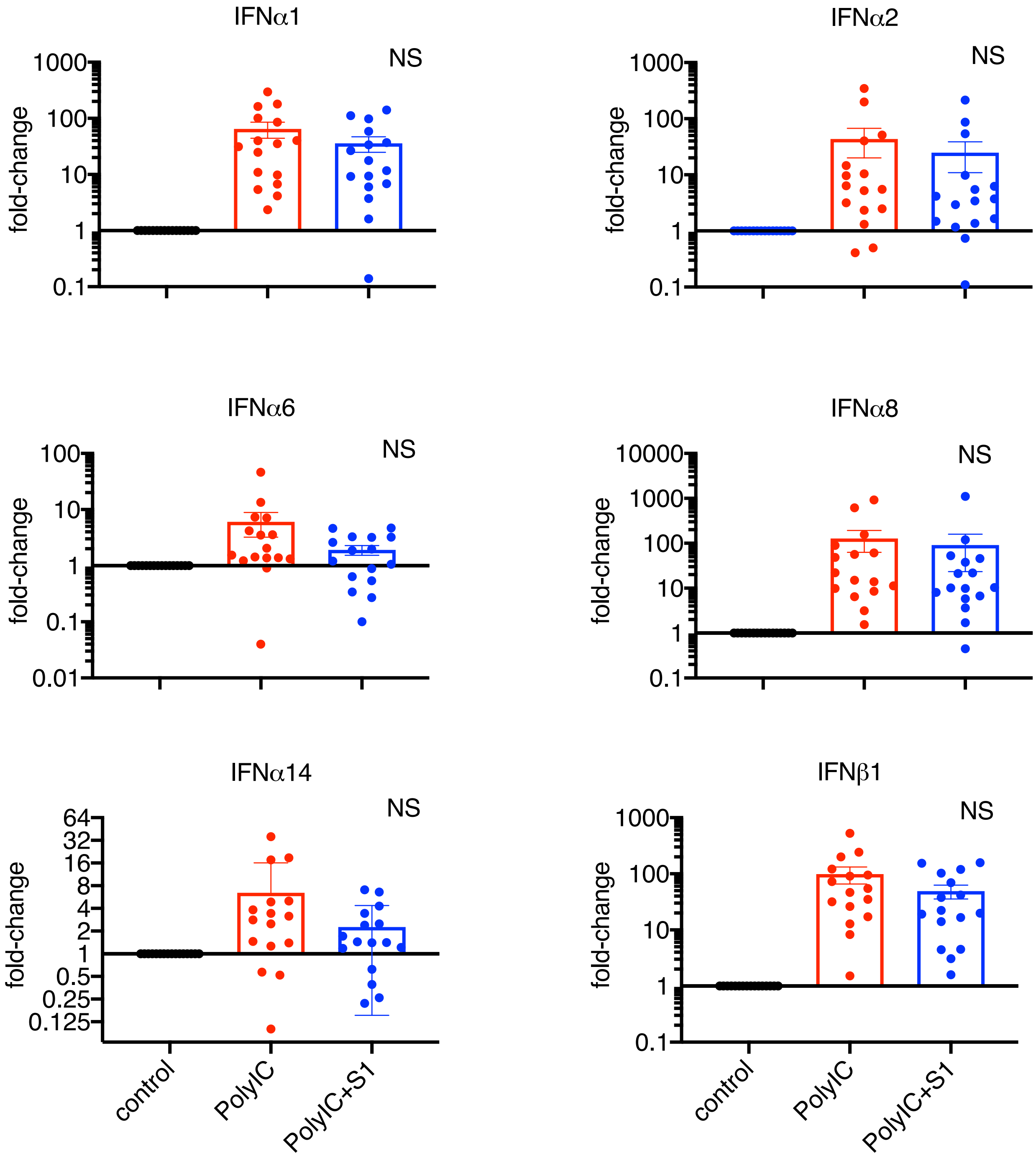

Supplementary Table 1. the correlations between expression levels of ACE2 and type I interferons in the presence of poly I:C only

**Poly I: C only**	**ACE2 vs. IFNa1**	**ACE2 vs. IFNa2**	**ACE2 vs. IFNa4**	**ACE2 vs. IFNa6**	**ACE2 vs. IFNa8**	**ACE2 vs. IFNa14**	**ACE2 vs. IFNb1**
Spearman r	0.14	0.01	0.25	0.54	0.14	0.22	-0.14
95% confidence interval	-0.40 to 0.60	-0.49 to 0.52	-0.29 to 0.67	-0.049 to 0.82	-0.40 to 0.60	-0.32 to 0.65	-0.60 to 0.39
P value (two-tailed)	0.61	0.96	0.35	*0.037	0.62	0.41	0.61

Supplementary Table 2. the correlations between expression levels of ACE2 and type I interferons in the presence of poly I:C and spike protein S1

**Poly I: C & S1**	**ACE2 vs. IFNa1**	**ACE2 vs. IFNa2**	**ACE2 vs. IFNa4**	**ACE2 vs. IFNa6**	**ACE2 vs. IFNa8**	**ACE2 vs. IFNa14**	**ACE2 vs. IFNb1**
Spearman r	0.54	-0.04	0.58	0.63	0.31	0.54	0.17
95% confidence interval	-0.04 to 0.82	-0.54 to 0.48	0.10 to 0.84	-0.18 to 0.86	-0.23 to 0.71	0.04 to 0.82	-0.37 to 0.62
P value (two-tailed)	*0.035	0.88	*0.02	*0.01	0.23	*0.035	0.53
